# Progress in oncolytic viruses modified with nanomaterials for intravenous application

**DOI:** 10.20892/j.issn.2095-3941.2023.0275

**Published:** 2023-11-24

**Authors:** Liting Chen, Zhijun Ma, Chen Xu, Youbang Xie, Defang Ouyang, Shuhui Song, Xiao Zhao, Funan Liu

**Affiliations:** 1 Department of Surgical Oncology and General Surgery, Key Laboratory of Precision Diagnosis and Treatment of Gastrointestinal Tumors, Ministry of Education, The First Affiliated Hospital of China Medical University, Shenyang 110001, China; 2 Phase I Clinical Trials Center, Ministry of Education, The First Affiliated Hospital of China Medical University, Shenyang 110102, China; 3 CAS Key Laboratory for Biomedical Effects of Nanomaterials and Nanosafety, CAS Center for Excellence in Nanoscience, National Center for Nanoscience and Technology, Beijing 100190, China; 4 Department of General Surgery, Panjin People’s Hospital, Panjin 124221, China; 5 Department of Hematology and Rheumatology, Qinghai Provincial People’s Hospital, Xining 810007, China; 6 State Key Laboratory of Quality Research in Chinese Medicine, Institute of Chinese Medical Sciences (ICMS), University of Macau, Macau 999078, China; 7 Center of Materials Science and Optoelectronics Engineering, University of Chinese Academy of Sciences, Beijing 100049, China; 8 IGDB-NCNST Joint Research Center, Institute of Genetics and Developmental Biology, Chinese Academy of Sciences, Beijing 100101, China

**Keywords:** Oncolytic virus, nanomaterials, drug delivery, tumor treatment, intravenous application

## Abstract

In oncolytic virus (OV) therapy, a critical component of tumor immunotherapy, viruses selectively infect, replicate within, and eventually destroy tumor cells. Simultaneously, this therapy activates immune responses and mobilizes immune cells, thereby eliminating residual or distant cancer cells. However, because of OVs’ high immunogenicity and immune clearance during circulation, their clinical applications are currently limited to intratumoral injections, and their use is severely restricted. In recent years, numerous studies have used nanomaterials to modify OVs to decrease virulence and increase safety for intravenous injection. The most commonly used nanomaterials for modifying OVs are liposomes, polymers, and albumin, because of their biosafety, practicability, and effectiveness. The aim of this review is to summarize progress in the use of these nanomaterials in preclinical experiments to modify OVs and to discuss the challenges encountered from basic research to clinical application.

## Introduction

In the past decade, tumor immunotherapy has made substantial progress in restarting and maintaining the tumor immune cycle, and activating the body’s anti-tumor response^1^. In oncolytic virus (OV) therapy, an essential branch of tumor immunotherapy, viruses replicate in and lyse tumor cells through various regulatory mechanisms, without affecting the growth of normal cells^2–4^. Currently, dozens of OV drugs have been developed for tumor treatment, including adenovirus (Ad), vaccinia virus, measles virus, poliovirus, herpes virus, retrovirus, reovirus, parvovirus H1, Newcastle disease virus, and coxsackievirus^5–8^. The administration methods have included primarily intratumoral injection, intravenous injection, intraperitoneal delivery, and hepatic artery infusion^2,6^.

Four OVs have been approved for clinical tumor treatment: Rigvir, T-vec, H101, and G47Δ^9–11^. Although the currently marketed products are administered locally, the OV administration route with the highest efficiency and safety in clinical trials is intratumoral injection, a route used primarily for surface tumors or localized tumors^6,12^. Clinically, OVs are used primarily to treat malignant tumors after failure of second- and third-line therapy; these tumors often exhibit metastases^13^. Although OVs injected into tumors are not transported to metastatic tumors, they can lead to the control of metastatic tumors (“distal effect”) by activating host antitumor immunity^14,15^. However, because therapeutic efficacy is limited, metastatic tumors remain difficult to cure. In addition, intratumoral injection has several insurmountable drawbacks: 1) the tumor tissue has highly elevated density and pressure, both of which limit successful entry of OVs into tumors^4,16^; 2) when a tumor grows deep in the human body, intratumoral injection is impossible^17^; 3) because of the abundant blood vessels in tumors, intratumoral injection may cause tumor rupture and enormous hemorrhage, and risks of tumor tissue shedding and metastasis may be present^18^; and 4) compliance is poor among patients who require industrial drug administration, particularly long-term medication^3^.

OV-associated clinical trials are increasingly testing various modes of administration, particularly intravenous injection^19^. However, the existing intravenous drugs still have many problems, such as poor drug stability, low targeting efficiency, low solubility, and adverse reactions^20–22^. Recent developments have led to increased use of nanotechnology in medicine, pharmacy, and biology. Nanoparticle delivery systems significantly increase the water solubility of drugs; prolong the half-life; and improve the loading of preparations, targeting, and biological safety^23^. In solid tumors, certain macromolecular substances are likely to penetrate into the tumor tissue and remain there for longer periods than in normal tissue, because of tumors’ abundant blood vessels, large vessel wall space, and lack of lymphatic circulation. This phenomenon has been termed the enhanced permeability and retention (EPR) effect.

In recent years, numerous preclinical studies have used various nanomaterials for drug delivery and have yielded promising results^24,25^. However, only several nanostructures have been approved for the clinical market to date^26^. On the one hand, this lack of approvals is because mice are the most commonly used animal models, and mouse solid tumors somewhat differ from those in humans. On the other hand, clinical translation inevitably requires large-scale production of anticancer drugs, and the price, safety, stability, and reproducibility of drug preparations of nanomaterials are critical^27,28^. To date, many nanoparticle-based medicines, primarily lipid, polymers, and albumin nanostructures, have been successfully applied in the clinical treatment of tumors^29^ (**Table 1**).

**Table 1:** Clinically approved liposomes, polymers, and albumin drugs

Name	Main NP ingredient (s)	Active pharmaceutical ingredient (s)	Approval (year)	Indication
**Liposome-based NPs**				
Doxil/Caelyx	Liposome; PEG	Doxorubicin	FDA (1995) EMA (1996)	Ovarian cancer; Kaposi’s sarcoma; osteomedullary melanoma
DaunoXome	Liposome	Daunorubicin	FDA (1996)	Kaposi’s sarcoma
AmBisone	Liposome	Amphotericin B	FDA (1997)	Systemic fungal infection
DepoCyt/DepoCyte	Liposome	Cytarabine	FDA (1999) EMA (2001)	Meningitis due to lymphoma
Myocet	Liposome	Doxorubicin	EMA (2000)	Breast cancer
Visudyne	Liposome	Vitipofen	FDA (2000) EMA (2000)	Wet senile macular degeneration
DepoDur	Liposome	Morphine sulfate	FDA (2004)	Postoperative pain
Mepact	Liposome	Mifenin	EMA (2009)	Osteosarcoma
Exparel	Liposome	Bupivacaine	FDA (2011) EMA (2020)	Postoperative pain
Marqibo	Liposome	Vincristine	FDA (2012)	Leukemia
Onivyde	Liposome; PEG	Irinotecan	FDA (2015) EMA (2016)	Pancreatic cancer
Vyxeos	Liposome	Daunorubicin/cytarabine	FDA (2017) EMA (2018)	Leukemia
Shingrix	Liposome	Recombinant herpes zoster vaccine	EMA (2018)	Herpes zoster; postherpetic neuralgia
Arikayce	Liposome	Amikacin sulfate	FDA (2018) EMA (2020)	Lung disease
Lipusu	Liposome	Paclitaxel	China (2006)	Breast, lung, and ovarian cancer
**Polymer-based NPs**				
Lupron Depot	PLGA	Leuprorelin acetate	FDA (1989)	Advanced prostate cancer
Adagan	PEG	ADA	FDA (1990)	ADA-SCID
SMANCS	Styrene maleic acid polymer	New oncomycin	Japan (1993)	Liver cancer; renal carcinoma
Oncaspar	PEG	ASNase	FDA (1994)	Acute lymphoblastic leukemia
Sandostatin Lar	PLGA	Octreotide acetate	FDA (1998)	Acromegaly
Trelstar	PLGA	Triptorelin	FDA (2000)	Acute lymphoblastic leukemia
Peglntron	PEG	Interferon α 2b	FDA (2001)	Hepatitis C
Pegasys	PEG	Interferon α 2a	FDA (2002)	Hepatitis B; hepatitis C
Neulasta	PEG	G-CSF	FDA (2002) EMA (2002)	Chemotherapy induced neutropenia
Somavert	PEG	HGH receptor antagonist	FDA (2003)	Acromegaly
Macugen	PEG	Anti-VEGF nucleic acid ligand	FDA (2004)	Neovascular age-related macular degeneration
Mircera	PEG	Erythropoietin β	FDA (2007)	Anemia associated with chronic kidney disease
Genexol-PM	mPEG-PLA	Paclitaxel	South Korea (2007)	Chemotherapy induced neutropenia
Cimzia	PEG	Anti-TNF Fab′	FDA (2008)	Crohn’s disease; rheumatoid arthritis; psoriatic arthritis; ankylosing spondylitis
Krystexxa	PEG	Uric acid	FDA (2010)	Chronic ventilation
Bydureon	PLGA	Exenatide synthetic	FDA (2012)	Type II diabetes
Andostatin Lar	PLGA	Paretotide	FDA (2014)	Acromegaly
Plegridy	PEG	Interferon β 1a	FDA (2014)	Recurrent multiple sclerosis
Movantik	PEG	Naloxone	FDA (2014)	Opioid induced constipation
Adynovate	PEG	Factor VIII	FDA (2015)	Hemophilia A
Triptodur Kit	PLGA	Triptorelin	FDA (2017)	Central precocious puberty
Sublocade	PLGA	Buprenorphine	FDA (2017)	Moderate to severe opiate use disorder
Palynziq	PEG	Phenylalanine ammonia lyase	FDA (2018)	Phenylketonuria
Jivi	PEG	Factor VIII	FDA (2018)	Hemophilia A
Eligard	PLGA	Leuprolide acetate	FDA (2002)	Prostate cancer
Zinostatin Stimalamer	SMA	NCS	Japan (1994)	Primary unresectable hepatocellular carcinoma
**Albumin-based NPs**				
Abraxane	Albumin	Paclitaxel	FDA (2005) EMA (2008)	Lung cancer; metastatic breast cancer; metastatic pancreatic cancer
Tanzeum	Albumin	GLP-1/HSA	FDA (2014)	Diabetes
Idelvion	Albumin	rIX-FP	FDA (2016)	Hemophilia B

In this review, we describe the advantages and disadvantages of lipids, polymers, and albumins as delivery vehicles (**Table 2**). We then describe related studies on the delivery of OVs by using lipid, polymers, and albumin (**Table 3**), and finally discuss their respective application challenges in the clinical treatment of tumors.

**Table 2:** Advantages and limitations of liposomes, polymers, and albumin in drug delivery

	Advantages	Limitations
**Liposome-based NPs**		
	Decreased systemic toxicity; improved tolerable dose of antitumor therapy; accelerated application of nanomedicine for site-specific delivery; improved drug utilization; unlimited packaging capabilities; relative ease of large-scale production; high-copy-number vector delivery	Short half-life and circulation time *in vivo*; Size too large (greater than 100 nm) to exploit the EPR effect; easy removal by the reticuloendothelial system after entering the body; single structural components; instability; complex sterilization process
**Polymer-based NPs**		
	Good biocompatibility; improved stability; prolonged circulation time; diminished adverse effects; adjusted physical and chemical properties; versatility	Limited amount; tumor accumulation rate likely to be low; weak tumor penetration; decreased transfection rate of drugs; low cytoplasmic drug release efficiency; difficulty in developing new drug delivery composite nanostructures
Synthetic polymer-based NPs	Excellent chemical versatility; batch-to-batch uniformity; hydrolysis without a need for enzymes; controlled release of drugs	Biological inertness; poor targeting
Natural polymer-based NPs	High biological activity; degradation by proteolysis through cell activation; controlled release of drugs	Strong immunogenic response; batch-to-batch variation;
**Albumin-based NPs**		
	Good biocompatibility; good stability; good drug loading performance; good targeting; longer half-life *in vivo*	Rapid degradation rate *in vivo*; decreased uptake of nanomaterials by tumor cells; relatively low stability

**Table 3:** Drug delivery of OVs by liposomes, polymers, and albumin

Oncolytic virus	Main NPs ingredient(s)	Therapeutic efficacy
Adenovirus	Liposome	Decreased production of adenovirus neutralizing antibodies (AdNAbs)
Adenovirus	DOTAP/DOPE	Improved antitumor effect
HSV	Liposome	Ready transfection into cultured cells; efficient production of infectious viruses; improved *in vivo* transduction efficiency
Reovirus	Cationic lipid	Promotion of reovirus delivery to the cytoplasmic matrix
M1	Soybean lecithin lipid	Blocking of viral immunogenicity; enhanced killing effect
Adenovirus	Anionic lipid	Enhanced transfection efficiency
Newcastle disease virus	iRGD lipid	Significant lysis of tumor and endothelial cells; significant promotion of antitumor immunity; significant inhibition of tumor neovascularization; reversal of the tumor suppressor microenvironment
Adenovirus	High molecular weight PEG (20–35 kDa)	Decreased liver accumulation
Adenovirus	PEG	Elevated resistance to inactivation by Ad-specific neutralizing antibodies (NABs); attenuation of the induction of innate antiviral immune responses
Vaccinia virus	PLGA	Appropriate inhibition of the growth of tumor volume
Adenovirus	ABPs	Markedly improved transduction efficiency of Ads
Adenovirus	CD	Markedly enhanced pathological effects in a dose-dependent manner
Adenovirus	pHPMA	Enhanced transduction; Prevention of hepatic sequestration of intravenously administered Ads
AAV	pHPMA	Enhanced protection against neutralizing antisera
Adenovirus	PEI	Elevated transduction efficiency; increased effectiveness of cancer cell killing
Adenovirus	PEI;PEG	Enhanced transduction efficiency; improved anti-tumor effect
Measles virus	PEI	Enhanced oncolytic activity
Adenovirus	Galactosylated polymers	Minimized adverse effects
Vaccinia virus	Amphiphilic polymer	Effective and significant decrease in the binding of anti-VV neutralizing antibodies
Adenovirus	PCDP	Accumulation and efficient replication in tumor tissues; improved anti-tumor effect
Adenovirus	PEG-b-PHF	Improved anti-tumor effect
Adenovirus	PPCBA	Enhanced pH sensitivity; improved release of enveloped OVs in the intracellular compartment of cancer cells
Adenovirus	Chitosan-PEG-FA	Diminished immune response; increased relative plasma circulating half-life; decreased hepatic accumulation (378-fold)
Adenovirus	PAMAM	Decreased immunogenicity during circulation; enhanced Ad circulation time
Adenovirus	Albumin-binding domain (ABD)	Protection of the viral capsid to overcome pre-existing NABs
Adenovirus	Albumin-binding domain (ABD)	Induction of 450-fold greater cytotoxicity in tumor cells than normal cells; decreased systemic toxicity; improved tumor targeting; NAB escape

## Liposomes

**Figure 1:**
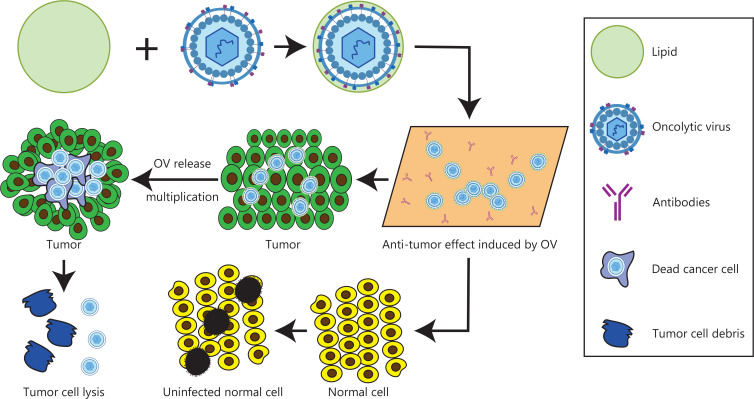
Schematic diagram of liposome encapsulated oncolytic virus.

In 1965, Zahednezhad et al.^30^ first proposed the idea of liposomes. In the 1970s, liposomes were widely recognized as a drug carriers^31^. In 1988, a liposome gel containing econazole was successfully marketed^32^. In 1990, injectable amphotericin B liposomes were listed in Europe^33^. Shortly thereafter, adriamycin liposomes were launched as the first cancer treatment drug liposome product in history. Currently, several liposome preparations have been marketed and widely used in clinical practice^34^, including daunorubicin liposomes, cytarabine liposomes, paclitaxel liposomes, and 5-fluorouracil heterophasic lipid plastids. Lipid materials have become the most widely used drug carriers, because of their advantages in encapsulating and transporting drugs^35^ (**Figure 1**). Liposomes are most commonly made of phospholipids and cholesterol, which are endogenous substances existing in organisms, with good histocompatibility and no immunogenicity^36^. The phospholipid molecules in the human body’s lipid bilayer each have a hydrophilic head made of phosphate and a hydrophobic tail made of 2 chain fatty acids, which confer both hydrophilicity and hydrophobicity^37^. Their internal hydrophilic environment makes liposomes suitable for encapsulating highly water-soluble drugs and gene fragments, whereas the middle of the bilayer, where the phospholipid tails are located, is a suitable location for storing and continuously releasing lipophilic drugs.

### Advantages and disadvantages of lipids as nanomaterials for drug delivery

**Figure 2:**
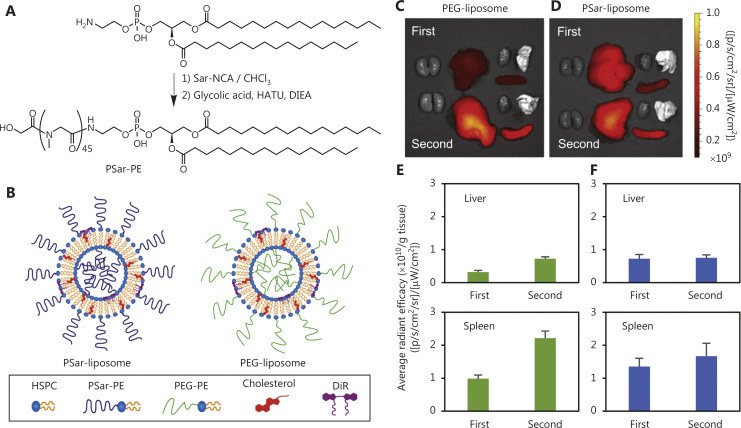
Modifying liposomes with PEG avoids clearance by the mononuclear phagocytic system (MPS) of the liver and spleen. Synthesis scheme for PSar (A) and schematic illustration of PEG- and PSar-liposomes (B). *Ex vivo* imaging of PEG-liposomes (C) and PSar-liposomes (D) in Wistar rats after the first and second injections. Images were obtained 4 h after injection. *Ex vivo* emission intensity of organs from Wistar rats with PEG-liposomes (E) and PSar-liposomes (F) 4 h after the first and second injections. Each bar represents the mean ± SD (*n* = 4–5). Copyright © 2020 Elsevier B.V. Sar-NCA, sarcosine-N-carboxyanhydride; HATU, 1-[bis(dimethylamino)methylene]-1H-1,2,3-triazolo[4,5-b]pyridinium 3-oxide hexafluorophosphate; DIEA, N,N-diisopropylethylamine; PSar, polysarcosine; HSPC, hydrogenated soya phosphatidyl choline.

The reasons for choosing liposomes as delivery carriers are as follows. 1) Dose control: Antitumor therapy can be administered at a dose that is more acceptable and with less systemic toxicity by using liposome-loaded medicines^38^. 2) Enhancing therapeutic effects: When liposomes are modified with hydrophilic polymers, such as polyethylene glycol (PEG), they are less likely to be removed by the liver’s and spleen’s mononuclear phagocyte system (MPS), thus effectively extending the liposomes’ *in vivo* circulation time and consequently increasing tumor enrichment. These aspects enable repeated dosing with liposome drug delivery systems^39,40^ (**Figure 2**). 3) Target delivery: After binding tumor cell target proteins, ligand modified liposomes enter cells through receptor mediated endocytosis^41^. The advent of liposomes responsive to stimuli including temperature, pH, and hypoxia has accelerated the application of nanomedicines for site-specific delivery^42^. 4) Arbitrary modification: Through internal or external physical, chemical, and biological stimuli, biological differences between tumor cells and normal tissues can be targeted. Modification of nanodrug carriers or molecular structures enables control of drug release timing and release rates, and improves drug utilization. 5) Additional advantages: Lipid-mediated drug delivery provides unrestricted packaging capacity, relative ease of mass production, and carrier delivery with high copy numbers^43^.

However, despite the clear advantages of drug delivery *via* liposomes, several problems exist with this strategy: 1) Excessive volume: Liposome-encapsulated viruses may become too large (greater than 100 nm) to exploit the EPR effect^44^. 2) Contagiousness of OVs: Because the infectivity of oncolytic adenoviruses (OAs) (including telomere scanning) largely depends on expression of the coxsackie and adenovirus receptor (CAR) in cells, and the attachment of the viral capsid, including the attachment of fibrin and viral receptors to the cell surface, the original telomerase-specific oncolytic adenovirus (TelomeScan) cannot produce cytotoxic effects in cancer cells with low CAR expression levels. TelomeScan (10 or 20 MOI) significantly inhibits the infectivity of HCT116 cells in culture medium supplemented with CAR antibodies. The presence of CAR antibodies had little effect on the ability of liposome-encapsulated plasmid DNA of telomerase-specific OAs (TelomeScan) to express GFP (Lipo-pTS). Because the CAR infectivity of Lipo-pTS is independent of, and different from, the original TelomeScan, our findings suggest that Lipo-pTS has the potential to infect and achieve cytotoxic activity, even in cancer cells with limited CAR expression on the cell surface^45^. 3) Single structural component: The reticuloendothelial system eliminates liposomes that enter the body, and the single structural component of liposomes is too simple compared to the biologically active cell structure^46^. 4) Instability: The instability of liposomes is also a difficult problem to solve, and is usually associated with the oxidation and hydrolysis of liposomes, drug leakage, and even liposome fusion^47^. 5) Difficulty in achieving sterile operations: The sterilization process also limits the application of liposomes; because of the tendency of liposomes to deteriorate or degrade after sterilization, suitable and effective sterilization techniques are necessary to ensure sterility^48^.

### Advances in lipid-encapsulated OVs

**Figure 3:**
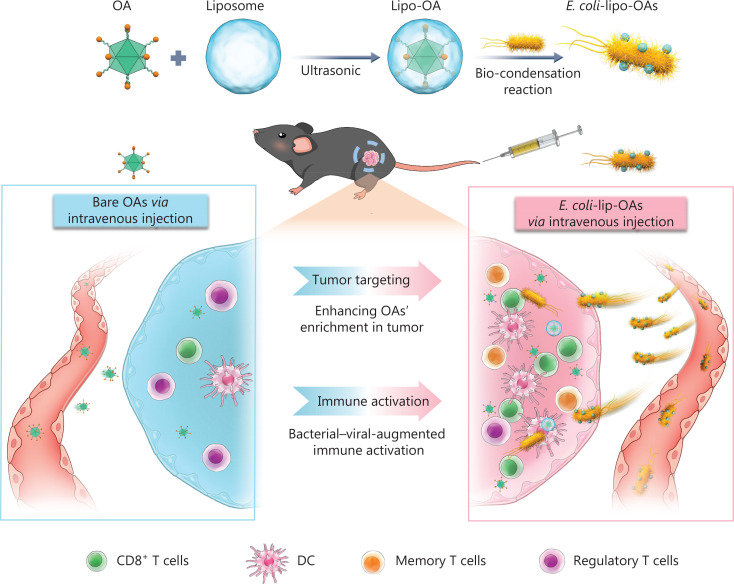
Schematic representation of self-propelled bacterium vessels carrying OAs that can home to tumor lesions and enhance antitumor responses through bacterial–viral-augmented immune activation. Copyright © 2022 American Chemical Society. DC, dentritic cell.

Antiviral antibodies have been found to exist in most of the population, and over 80% of adults possessing anti-Ad5 antibodies^49^. The first problem encountered with intravenous OVs is attack by neutralizing antibodies. As early as the 1990s, liposomes were used as carriers of OVs to ward off attacks from neutralizing antibodies. To fight against Ad-specific neutralizing antibodies (AdNABs), Katsuyuki et al.^45^ have studied the potential of TelomeScan plasmid DNA encapsulated in liposomes capable of expressing GFP (Lipo-pTS) to function as an oncolytic adenoviral agent for systemic delivery. In HCT116 colon cancer cells, lipo-pTS with a diameter of 40–50 nm has been found to have anti-tumor efficacy, as well as tumor specificity unrelated to CAR. In addition, intravenous lipo-pTS decreases the generation of AdNABs and retains high cytotoxicity even when AdNABs are present^45^. Fu et al.^50^ have prepared 3 different forms of HSV vectors: HSV capsids, complete viral particles, and purified viral DNA. All 3 types of HSV can easily produce contagious viruses when transfected into cultured cells, because intravenous liposome delivery of HSV vector DNA successfully avoid host immune neutralizing antibodies. Moreover, liposome-cloaked oncolytic Ad (oAd) conjugated to tumor-homing *Escherichia coli* BL21 (designated *E. coli*-lipo-oAd) has been found to enhance cancer immunotherapy^51^ (**Figure 3**).

In recent years, several studies have shown that liposome-encapsulated OVs significantly increase the antitumor ability of OVs. Among them, cationic liposomes has become a major topic. Kwon et al.^52^ have complexed negatively charged oncolytic Ad genomic DNA with cationic liposomes (DOTAP/DOPE) to generate pmT-d19/stTR+DOTAP/DOPE lipoplexes, which have been found to achieve comparable cell death to that induced by oAd after treatment with liposomes. Therefore, Ad genomic DNA in liposomes can efficiently generate active oncolytic Ad progeny. In addition, systemic administration of pmT-d19/ stTR + DOTAP/DOPE liposomes have been found to elicit an extremely effective antitumor response *in vivo*, resulting in a 90.5% and 92.4% lower tumor volume than observed after bare-colytic Ad or bare-colytic Ad genomic plasmid therapy, respectively. The antitumor effect of oncolytic Ad DNA liposomes is better than that of naked oAd and naked oAd genome plasmids^53^. Sakurai et al.^54^ have complexed reovirus with cationic liposomes as a transfection agent. Reovirus cationic liposome complexes (reoplex) and reovirus alone both showed equivalent levels of tumor cell killing efficacy in reovirus-sensitive tumor cells, whereas the degree of tumor cell killing activity in resistant tumor cells was more than 30% higher than that of reovirus alone. Receptor-mediated tumor cell death was successfully induced in tumor cells that had previously received cathepsin inhibitor treatment. Interferon (IFN)- and apoptotic gene mRNA levels markedly increased during repeated compounding. According to these findings, cationic liposomes effectively encourage the transfer of reoviruses to the cytoplasmic matrix and subsequent induction of apoptosis.

However, after entering the body, cationic liposomes absorb negatively charged serum proteins and consequently cause liposome nanomaterials to aggregate, thus significantly decreasing their ability to penetrate tumors *via* the EPR effect^44^. In eukaryotic cell membranes, anionic liposomes have the benefits of being less immunogenic and hazardous than cationic liposomes. In addition, anionic liposomes can undergo a phase change when calcium ions are present, thereby aiding in the electrostatic adsorption process through which liposomes encapsulate OVs^55^. In addition, Wang et al.^56^ have used the membrane hydration method to encapsulate M1 into soybean lecithin liposomes; these liposomes effectively prevented M1 neutralizing antibody from binding M1 without affecting viral infectivity, thereby blocking the immunogenicity of the virus and enhancing its killing effect on colon cancer (LoVo) and human hepatoma (Hep 3B) cells. Zhong et al.^55,57^ have reported a calcium-induced phase transition approach to encapsulate Ad5 in anionic liposomes (Ad5-AL). Gene expression was 6-fold higher in adenocarcinoma cells infected with Ad5-AL than in cells infected with the bare virus. In addition, an antibody neutralization experiment revealed that Ad5-AL was more efficient than the complex of Ad5 and cationic liposomes (Ad5-CL) in shielding Ad5 from neutralization, because it inhibited both naked Ad5 and Ad5-CL at higher dilutions. This finding may have 2 explanations. First, the Ad5-AL group had higher gene expression; second, a Ca^2+^ chelator increased Ad5 uptake and gene expression. Thus, these findings demonstrate that anionic encapsulation in liposomes enhances the transfection efficiency of Ad5 and significantly increases gene expression in mouse airway tissues when delivered by intratracheal instillation *in vivo*^58^.

### Application prospects

Broad applications and substantial development possibilities exist for OV encapsulation and delivery to tumor locations by using lipids. Modified lipid drug delivery systems enable a range of functions, such as boosting therapeutic efficacy, lowering systemic adverse responses, avoiding drug resistance, destroying tumor stem cells, and penetrating biological barriers. Therefore, these lipids are worthy of research and development. The theoretical study of lipids is becoming more complex, and research on laboratory lipid preparation techniques is maturing. Lipids can be used in a variety of methods and applications, whether systemic or topical, to enable targeted drug delivery that effectively deposits drugs at lesion sites, thus achieving better effects while minimizing unwanted responses. However, the industrialization of lipid nanoparticle drug delivery systems continues to pose barriers to their advancement, such as onerous quality control procedures and exorbitant pricing for membrane components including injection-grade phospholipids. Lipid drug delivery systems will have excellent development prospects as science advances, and pharmaceutical devices and technologies are regularly updated.

## Polymers

**Figure 4:**
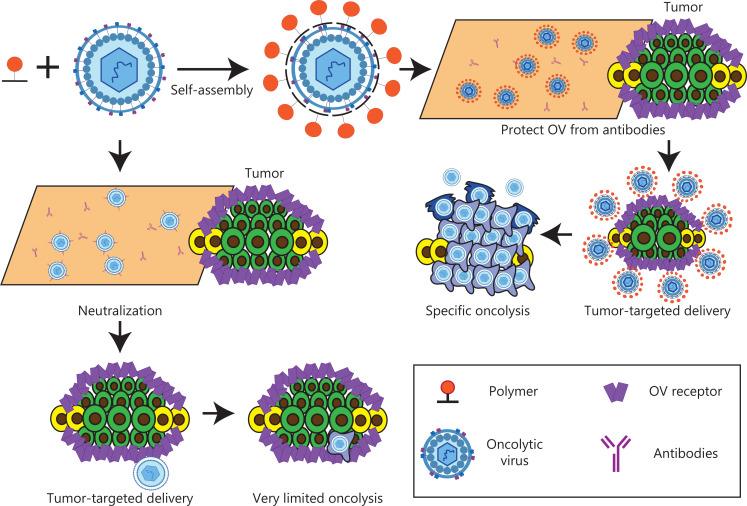
Schematic diagram of polymer-encapsulated oncolytic virus.

A polymer is a substance with a high molecular weight that is composed of several identical and simple structural units linked repeatedly by covalent bonds. Because of their ease of synthesis, polymer nanostructures have a wide range of applications in multiple aspects of nanomedicine. First, polymer nanomaterials can be directly used as nanomedicines for the treatment of diseases; the most famous example is Copaxone^®^, a synthetic peptide compound. In 1996, Copaxone^®^ was first approved for marketing in Israel and in the same year was approved by the US FDA for the treatment of multiple sclerosis^59^. However, polymer nanomaterials are also commonly used to deliver drugs. Polymers and drugs are linked by chemical bonds. After entering the body, chemical bonds are broken by endogenous or exogenous changes, and the drug is released at the target site (**Figure 4**). In the 1960s and 1970s, polymers were developed for biodegradable sutures. Since then, because of polymers’ excellent degradability and bioavailability, the field of drug delivery has flourished^60^. In recent years, polymer nanomaterials with targeted controllable release have been used to encapsulate drugs; *in vitro* laboratory research and increasing clinical trials have already led to the approval of several products on the market^61^. Polymers are applied in 2 main drug types. The first type is polymer-drug conjugates, which are used to increase the bioavailability and half-life of drugs. One representative nanomaterial is PEG, in the drug PLEGRIDY^®^, which was approved by the FDA in August 2014 for the treatment of adults with relapsing-remitting multiple sclerosis^62^. Another representative drug, adynovate, was approved in 2015 for the treatment of pediatric hemophilia A in patients under 12 years of age^63^. The second type of polymeric drug uses the degradability of polymers to achieve controlled drug release. A representative nanoparticle is poly-(lactic-co-glycolic acid) (PLGA), and the representative drug Eligard^®^ is commonly used to treat advanced prostate cancer^64^.

### Advantages and disadvantages of polymers as nanomaterials for drug delivery

Overall, the main advantage of using polymers as drug carriers is their biodegradability. Biodegradable polymer drug carriers can be broken down in the body to produce nontoxic natural byproducts, such as carbon dioxide and water. Biodegradable polymers can divided into 2 categories, synthetic and natural, each with its own advantages and disadvantages^65^. Synthetic polymers, for example, are characterized by their precisely controlled chemical structures and biological inertness. Therefore, they exhibit excellent chemical versatility and batch-to-batch uniformity^66^. However, the development of synthetic polymers is also limited by their biological inertness^67^. In addition, an essential topic in the field of polymer science is enhancing the interaction between polymers and target cells, mainly by incorporating functional materials into the structures of synthetic polymers^68^. In contrast, unlike natural polymers, synthetic polymers can be hydrolyzed without a need for enzymes^69^. Natural polymers have 3 major characteristics: strong biological activity, excellent biocompatibility, and degradation by proteolysis through cell activation^70^. However, higher biological activity leads to a stronger immunogenic response. Furthermore, natural polymers are obtained from natural resources, and their chemical structures are difficult to purify, characterize and identify accurately, thus leading to batch-to-batch variability. These 2 major issues have hindered further development^71^.

Another substantial advantage of polymer drug carriers is the controlled release of drugs^72^. The endogenous controlled release mechanism of polymers can be summarized as follows: 1) drug diffusion through water-filled pores; 2) osmotic pumps; 3) drug diffusion through the polymer matrix; and 4) substantial erosion. The exogenous controlled release mechanism triggers release through structural changes caused by endogenous and exogenous stimuli (for example, polymer surface layer shedding, charge conversion, and degradation). Stimuli include temperature, light, specific biomarkers (such as urea and glucose), redox potential, and any combination thereof (such as pH/temperature, pH/magnetic field, and so on)^73^. Polymer gels and polymer micelles are common vectors for achieving exogenous controlled release responses with drug delivery systems.

Polymer-based drug carriers also have advantages, such as improved stability, prolonged circulation time, diminished adverse effects, adjusted physical and chemical properties, and versatility (with payloads as diverse as anticancer drugs and gene therapy reagents)^74^. Although polymers have numerous advantages as drug carriers, they still face at least 6 major obstacles that must be overcome to enable extensive clinical application: 1) drug loading is limited in comparison to that of more common drug carriers, such as liposomes; 2) the drug’s tumor accumulation rate is most likely to be modest, because of subpar vascular extravasation and other factors; 3) tumor penetration is weak (the tumor has dense extracellular matrix and elevated interstitial fluid pressure); 4) partial polymer materials decrease the transfection rate of drugs, such as PEG; 5) the cytoplasmic drug release efficiency is low, because of encapsulation in the acidic endosome/lysosome compartment; and 6) for some natural polymers, determining the material’s composition and assessing its biological effects are difficult, thus hindering development of drug delivery composite nanostructures^75–77^.

### Advances in polymer-encapsulated OVs

#### PEG

**Figure 5:**
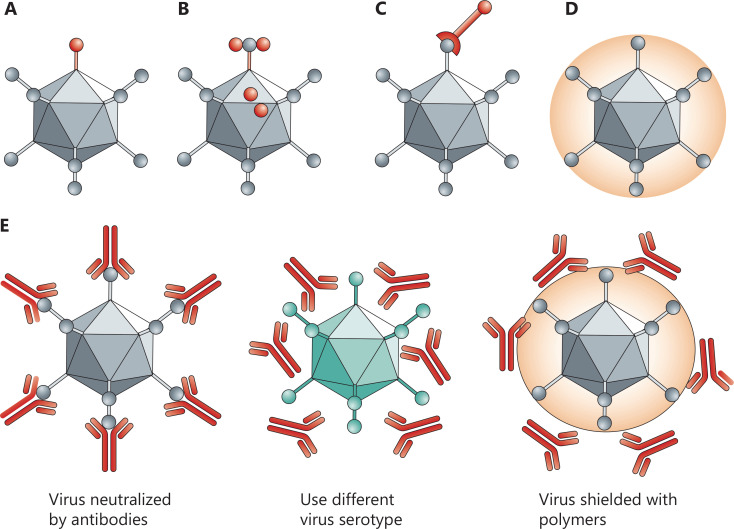
Biochemical *vs.* genetic modification of viruses. In genetic retargeting, an exogenous ligand is fused to, or replaces, the normal receptor-binding protein of the virus. (A, B) Viral particles can be modified chemically. (C–E) Shielding the virus against antibodies. Pre-existing neutralizing antibodies in humans can interfere with efficacy. Changing virus serotypes and coating particles with shielding polymers can address the neutralizing-antibody problem. Copyright © 1969, Springer Nature.

PEG, a hydrophilic linear polymer, has biocompatible and nonimmunogenic characteristics^78^. These properties make PEG the polymer of choice for the delivery of OVs^79^ (**Figure 5**). The amount of surface modification and the molecular weight of PEG significantly affect the amount of PEGylation that can be achieved for OVs^80,81^. Ad modification with low molecular weight PEG (2–5 kDa), for instance, does not decrease viral accumulation in the liver^80^. In contrast, liver accumulation decreases when Ad is modified with slightly higher molecular weight PEG (20–35 kDa)^82^. Accelerated blood clearance (sometimes referred to as the “ABC phenomenon”) is a unexpected immunogenic reaction seen in PEG conjugates that causes rapid clearance of PEG-based nanocarriers. After repeated administration, the widely documented ABC phenomenon decreases the potency of PEG conjugates and nanocarriers. C activation-associated pseudoallergy, another unanticipated immune response, significantly decreases the safety of PEG-based nanocarriers and has been associated with a decline in the efficacy of PEG-based therapy in clinical trials. One important structural component affecting immunological safety is PEG length. Long and short chain PEG conjugates are relatively more likely to cause the ABC phenomenon, because this effect is biphasic. PEG density exhibits a biphasic effect similar to that of PEG length. However, PEGs with diminished ABC phenomenon have been identified among lower and higher density PEGs. Yao et al.^83^ have reported that PEGylation with 45% coverage using 20 kDa PEG, targeting reactive amines, yields the optimal PEGylation outcome for Ad.

O’Riordan et al.^84^ have demonstrated that PEGylated Ad is more resistant to inactivation by AdNABs than bare Ad. The covalent bond between PEG and the adenovirus surface is achieved primarily through the use of activated PEG trimethyl monomethoxy PEG, which preferentially binds lysine residues in an ε amino terminal reaction. The components in the capsid that trigger neutralizing immune responses—i.e., hexanone, fiber, and pentosan bases—are also the main targets of PEG formation, thus enabling PEG to effectively protect capsid proteins. Furthermore, Croyle et al.^85^ have shown that PEGylation of Ad successfully avoids NABs. However, because of the tendency of PEGylated Ad to accumulate and precipitate, these studies are currently applicable only to intratracheal injection. Wilson et al.^86^ have demonstrated that systemically administered naked Ad results in greater adaptive immune responses than PEGylated Ad. Specifically, bare Ad induces greater Ad-specific NAB production than PEGylated Ad. PEGylation of Ad decreases interleukin (IL)-6 levels in the serum after systemic delivery, thus blunting the development of innate antiviral immune responses^87–89^. PEGylated Ad markedly decreases hepatotoxicity and innate antiviral immune responses, but this shielding may diminish oncolytic efficacy after intravenous injection therapy^90^.

Despite the substantial benefits of PEGylation in improving systemic delivery of Ad, removing Ad’s ability to attach to CARs has been shown to weaken several essential components that support its higher transduction efficiency^91^. Ad undergoes a two-step process for cellular entry. Initially, Ad fibers bind CAR, and this is followed by engagement of cellular integrins (αvβ3 and αvβ5) and arginine glycine aspartate motifs (RGD) on the penton base of Ad. Additionally, the interaction of Ad5 vector particles with plasma proteins, such as coagulation factor X (FX), which binds the main Ad5 capsid protein hexon, significantly affects the distribution of Ad5 vector particles in living organisms^92^. Interestingly, PEGylated Ad decreases cellular internalization and hampers virion trafficking to the nucleus, with assistance from microtubules^93^. Therefore, additional investigations are required to address the restrictions of PEGylated Ad. Choi et al.^94^ have used PEG-poly(N-[N-(2-aminoethyl)-2-aminoethyl-2-aminoethyl]-L-glutamic acid copolymers (PEG-PLNG) to create a sequence of 6 biocompatible polymers, each with a distinct PEG molecular weight and amine group count (2 or 5). In general, the relationship between the polymers’ surface charge and size and amine content was inverse. The need for more amine groups within the polymer was demonstrated by the higher transduction efficiencies observed for Ad nanocomplexes made of PEG-PNLG variants with 5 amine groups in both CAR-positive and CAR-negative cells than Ad complexes made of PEG-PLNG with 2 amine groups^94^.

#### PLGA

**Figure 6:**
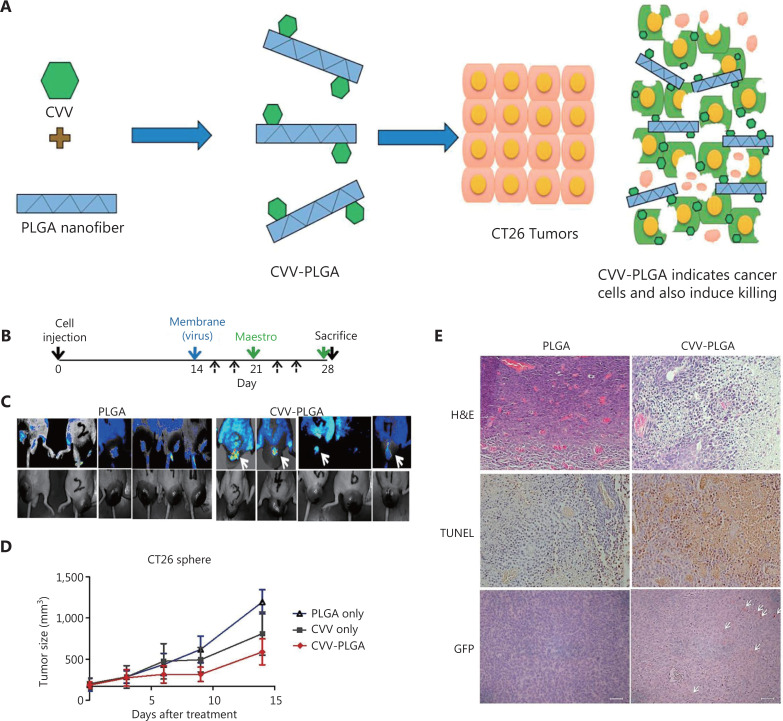
CVV-PLGA nanofiber. (A) Diagrammatic sketch of the use of CVV-PLGA in tumor therapy. (B–E) Effects of treatment with CVV-PLGA on CT26 tumors in BALB/c nude mice. Copyright © 2018 Elsevier B.V. H&E, hematoxylin and eosin; GFP, green fluorescent protein.

PLGA, a copolymer comprising lactic acid and glycolic acid, is recognized as a biocompatible and biodegradable material^95^. Commonly referred to as a “smart polymer,” PLGA first found application in the development of absorbable surgical filaments during the early 1970s^96^. Over the past few decades, its utility has expanded, and it is currently among the most successful drug delivery systems, because of its remarkable properties. Its advantages include biocompatibility, sustained release capability, non-toxicity, non-immunogenicity, and an ability to accommodate a wide range of drugs with polymer-friendly adaptability^97–99^. Notably, the hydrolysis of PLGA yields endogenous compounds that are easily metabolized. Furthermore, PLGAs can be processed into amorphous or crystalline forms that vary in shape and size, thereby facilitating the encapsulation of diverse molecules, both hydrophobic and hydrophilic^100,101^. Badrinath et al.^102^ have embedded a cancer-favoring oncolytic vaccinia virus (CVV) on PLGA nanofibrous membranes and compared the therapeutic effects of empty PLGA nanofibrous membranes and CVV-PLGA nanofibrous membranes. Within 48 hours, anticancer activity of CVV was observed, on the basis of continuous CVV release from the PLGA nanofiber membrane, while the anticancer activity of CVV is correctly preserved during the embedding process. Studies using tumor xenografts in living organisms demonstrated that the CVV liberated from the PLGA nanofiber membrane was effectively transported into tumor tissue. Therefore, these findings indicate that although the membrane itself does not effectively restrict the growth of tumor volume, the PLGA nanofiber membrane implanted with CVV does (**Figure 6**).

#### Cationic polymers

Cationic polymers are promising carriers for viral gene delivery. Through ionic complexation, these polymers form nanoscale assemblies with genetic materials^103–105^. Integration of cationic polymers with viral vectors has been used to bolster their gene transfer efficiency. After complex formation, the surface charge of the therapeutic agent shifts from negative to positive, thus facilitating heightened interactions with anionic cell membranes and promoting cellular uptake of the therapeutic drug^106^.

##### Arginine

Recently, gene delivery techniques using arginine-grafted biorepeatable polymers (ABPs) have been created^107^. Because arginine is typically present in protein transduction domains and cell-penetrating peptides, adding it to Ads can markedly increase their transduction efficacy^108^. ABP also has reducible disulfide bonds in its backbone, which enable it to biodegrade by shrinking the cytoplasm, thus effectively releasing Ads from the multiplex and decreasing cytotoxicity. ABP-coated Ad complexes have been shown to have diminished toxicity and improved transduction efficiency in both CAR-positive and CAR-negative cells. Additionally, unlike bare Ad, the ABP-coated Ad complex’s activity is not considerably decreased by serum, and innate immunological reactions and hepatotoxicity are both greatly mitigated. Decreased liver sequestration and increased anticancer effectiveness have been observed when cationic polymers or other hydrophilic polymers (such as PEG) are chemically coupled^109^.

##### pHPMA

pHPMA(poly-[N-(2-hydroxypropyl) methacrylamide]), another cationic polymer, has been extensively used for conjugation with OVs. Wang et al.^110^ have synthesized a series of pendant cationic oligolysine with varying lengths (K5, K10, and K15) through charge interactions for coating Ad5. Among these formulations, pHK10 exhibited superior transduction efficiency to the other 2 variants. Transduction tests conducted in CAR deficient Chinese hamster ovary cells indicated that pHK10 Ad5 virus, compared with Ad5, increased the transduction efficiency of cell entry. The cationic pHK10 formulation defeated Ad dependent CAR mediated cell entry, a finding attributed to the oligosaccharides found in HPMA. The main internalization pathway of polymer coated Ad5 was demonstrated to redirect from CAR to sulfated proteoglycans. Therefore, this straightforward viral modification technique may potentially provide a remedy for enhancing Ad5 conversion to a variety of CAR negative cell types.

To promote effective liver detoxification and decrease Ad neutralization by NABs, Fisher et al.^111^ have attached pHPMA to the Ad surface. Approximately 74% of the amine groups on Ad were altered by pHPMA, according to *in vitro* research. The Ad interaction with NABs decreased by almost 80% as a result of these changes. The ability of pHPMA to stop hepatic sequestration after intravenous delivery of Ads was highlighted by the observation that the liver transgenic expression levels after intravenous administration of pHPMA-modified Ads were 10,000 times lower than those of naked virions^112–115^.

Prill et al.^116^ have used pHPMA polymers activated with maleimide groups or pyridyldithio groups to examine the effects of coating Ad with a “bioresponsive” shield. According to their research, the addition of an irreversible coating affected how the virus was transported inside the cell to the nucleus. The bioactive covering did, however, eventually permit particle movement. The effects of the charge of Ad’s reactive polymer coating were then examined *in vivo*, and a positively charged reactive polymer coating was found to increase the likelihood of liver infection.

Carlisle et al.^117^ have applied a coating to Ads by using pHMPA-based polymers with bioreactive side chains containing amine-reactive TT groups and acid-labile hydrazone. *In vivo* tests showed a 50-fold greater circulation half-life and 8,000-fold lower hepatic accumulation than observed with naked Ad. Through ultrasound-assisted delivery, the tumor accumulation of the coated virus was improved, owing to its favorable circulatory profile. Combination of polymer-coated Ad, gas bubbles, and focused ultrasound enhances tumor infection by at least 30 times. In addition, the infectivity was 40-fold greater than that observed in controls without ultrasound, at distances more than 100 mm from the vasculature.

Furthermore, Carlisle et al.^118^ have examined how pHPMA coating affects other adeno-associated viruses (AAV) beyond Ad5. They discovered that using an established, effective adenovirus encapsulation method to combine reactive HPMA copolymers with AAV5 did not decrease AAV5 infection, because the presence of free polymers in fact promoted viral infection. An additional study has revealed that the increased activity induced by free polymers is mediated through typical infection mechanisms and has some resistance to neutralizing antibodies. While this observation was essentially an experimental artefact, the phenomenon might be applied to stop viruses from spreading locally in the environment.

##### PEI

Polyetherimide (PEI), which has remarkable cellular uptake and endosomal escape properties, has long been regarded as the gold standard for gene delivery^119^. However, the cytotoxicity of 25 kDa PEI substantially restricts its therapeutic use^120–122^. A polydegradable, bioreductible, core-crosslinked PEI (rPEI) copolymer with different molecular weights (16 and 32 kDa) has been created by Choi et al. and coated on the surfaces of OAs. Ad complexed with 16 kDa rPEI has shown greater transduction efficiency and cancer cell killing efficiency than naked Ad in both CAR-positive and CAR-negative cells^123^. A variety of cancer cells can be treated, because this increased anticancer cytotoxicity is more readily apparent in CAR-negative MCF7 cells. A549 and HT1080 cancer cells treated with Ad/16 kDa rPEI also display considerably lower Met and vascular endothelial growth factor (VEGF) expression than naked Ad or Ad/25 kDa PEI. These results indicate that the combination of shMet expressing oncolytic Ad and biodegradable co-crosslinked PEI can serve as an effective and safe cancer gene therapy. A polydegradable cationic carrier based on the mPEG-PEI-g-Arg-S-Arg-S-Arg-g-PEI-mPEG (Ad/PPSA) copolymer was developed by Jung et al.^124^ for coating Ad. The measurement of Ad/PPSA particle size demonstrated that the overall size and cationic charge increased with increasing polymer concentration. Complexing Ad with PPSA resulted in a 2.24-fold increase in anticancer activity in MCF7 tumor xenografts. Intravenous injection of DWP418/PPSA led to lower innate immune responses than naked Ad, because it released interleukin-6 cytokines into the serum. DWP418/PPSA’s vector targeting to CAR negative and positive cells, and its improved anti-tumor effects, indicated the promise of this tool in cancer gene therapy. Nosaki et al.^125^ have gradually added cationic PEI and anionic chondroitin sulfate to form an oncolytic measles virus (MV) multimolecule. The polymer-coated virus displayed greater oncolytic activity *in vitro* than the bare the genetically engineered measles virus (MV-NPL) in the presence of anti-MV neutralizing antibodies. Additionally, animals treated with the polymer-coated MV-NPL showed greater complement-dependent cytotoxicity and antitumor activity than mice treated with the bare virus. This new polymer-coated MV-NPL is anticipated to contribute to clinical cancer treatment in the future^126^.

#### Passive-targeted polymer

Complexation with polymers containing targeting moieties can be used to improve preferential tumor accumulation of systemically delivered OVs. Relaxin (RLX)-expressing oncolytic Ads (oAd/RLX), which break down the dense tumor extracellular matrix in highly desmoplastic pancreatic cancer, have been complexed with biodegradable polymer [poly(ethyleneimine)-conjugated poly(CBA-DAH); PCDP]. This combination allows human bone marrow-derived mesenchymal stromal cells to integrate more effectively than weakly loaded naked Ads^127^. Ad/RLX-PCDP-loaded human bone marrow-derived mesenchymal stromal cells have shown effective Ad uptake and proliferation after systemic treatment. In an orthotopic pancreatic tumor model, our method achieved stronger anticancer effects than observed with bare Ad by increasing tumor cell death due to apoptosis, as well as decreasing the tumor extracellular matrix. 

Using a U87 tumor xenograft mouse model, Choi et al.^128^ have created the pH-sensitive block copolymer, methoxy poly(ethylene glycol)-b-poly(l-histidine-co-l-phenylalanine) (PEG-b-PHF) to target the acidic tumor microenvironment and limit tumor growth. They have investigated tumor-targeting capabilities by complexing pH-sensitive polymers encoding an oAd transcriptional inhibitor of the VEGF promoter (KOX). The cancer cell killing effects of KOX/PEG-b-PHF (pH 6.4) were greater than those of naked KOX and KOX/PEG-b-PHF (pH 7.4), thus suggesting more effective antitumor activity. Inspired by this method, the authors produce a PEG-b-pHis/Ad complex by physically complexed Ad with a pH-sensitive block copolymer, methoxy poly(ethylene glycol)-b-poly(l-histidine) (mPEG-b-pHis) under acidic pH conditions in tumor tissue^129^. The PEG-b-pHis/Ad complex considerably outperformed naked Ad in terms of conduction efficiency at pH 6.4, and showed significantly greater therapeutic efficacy in both CAR-positive and CAR-negative tumor types.

With the goal of covering the Ad surface, Moon et al.^130^ have created a pH-sensitive and bio-reducible polymer (PPCBA). The Ad-PPCBA complex responded to pH, displaying greater cellular absorption at pH 6.0 and improved cellular uptake in both CAR-positive and CAR-negative cancer cells at pH 7.4. Importantly, in a human xenograft tumor model, intratumoral and intravenous treatment with Ad-PPCBA nanocomplex expressing VEGF-specific shRNA resulted in a 3-fold greater anticancer effects than naked Ad, with lower levels of systemic toxicity. According to these results, adding bioreducible linkers improves pH sensitivity and increases envelope OV release within the intracellular space of cancer cells.

#### Tumor-targeted polymers

In addition to passive targeting, the incorporation of tumor-targeting motifs actively targets several receptors that are overexpressed in tumor tissue. The first research demonstrating successful tumor-targeting OVs used a folate (FA)-anchored Ad/chitosan-PEG-FA nanocomplex created by Park et al.^131^. To prevent impairment of Ad function, the ionically cross-linked chitosan layer on the Ad surface offers a site for chemical coupling with PEG and subsequent binding to FA (targeting moiety at the end of heterofunctional PEG). In comparison to bare Ad, PEGylation *via* the chitosan amine group caused a lower immunological response and an extended plasma circulation half-life. Ad/chitosan-PEG-FA nanocomplexes, compared with bare Ad, resulted in 75.3% lower Ad-specific NAB synthesis in mice; moreover, the hepatic accumulation was 378-fold lower^132^. Additionally, after subcutaneous administration of a systemic dose of Ad/chitosan-PEG-FA nanocomplex, mice with KB tumors expressing the folate receptor (FR) showed a 285-fold greater intratumoral viral load than observed with bare Ad. These results suggest that, by attaching polymers with appropriately localized moieties to the surface of OVs, tailored distribution of OVs can be accomplished without compromising viral infectivity.

Dendrimers made of polyamidoamine (PAMAM) have good water solubility, are not immunogenic, and have surface functional groups that are easily modified with drugs and targeted ligands. PEGylated PAMAM dendrimers (PPE) and EGFR-specific Erbitux antibodies have been used by Yoon et al.^133^ to create complexes with Ad. Because of steric hindrance, complexation with PEG based PAMAM (PP) dendrimers inhibits Ad transduction. In an EGFR-positive orthotopic lung tumor model, systemic injection of OA/DCN-shMet/PPE has been found to result in a remarkable 14.9-fold suppression of tumor growth with respect to naked Ad, thus causing total tumor regression in 66% of treated animals. A 290-fold increase in viral genomes found in the blood after 24 hours by pharmacokinetic profiling suggests that ErbB coupled and PEG functionalized PAMAM dendrimers can effectively mask the capsid of Ad and delay the effective internalization of oncolytic Ad into EGFR positive tumors, while decreasing the toxicity of systemic administration of nude oncolytic Ad^134^.

#### Different polymers

Biodegradable poly (cystaminoacrylamide-diaminohexane) [poly (CBA-DAH)] (CD) has been studied as a polymeric vehicle for oncolytic Ad dispersion. When Ad/CD-PEG-RGD is physically compounded with RGD peptide-coupled CD polymer (CD-PEG-RGD), the pathogenic effect of oncolytic Ad releasing shRNA against IL-8 has been found to be dramatically and dose-dependently amplified, as compared with the cell specificity of naked Ad in cancer cells. RGD-conjugated polymers also send Ads to cells that express certain integrins, regardless of CAR status^135^. Ad/CD-PEG-RGD treatment decreases the expression of IL-8 and VEGF while increasing apoptosis in the cells.

Garofalo et al.^136^ have used galactosylated polymers as carriers for systemic delivery of OAs in hepatocellular carcinoma cell lines. The results have demonstrated that the use of computational polymers for coating and targeting is a safe and effective therapeutic strategy with minimal adverse effects. These findings provide a basis for future studies using OV complexed with polymers for specific and more efficient targeting of hepatocellular carcinoma.

Hill et al.^137^ have coated vaccinia virus (VV) with an amphiphilic polymer, and attached an antibody-targeting anti-mucin-1 (aMUC1), thus yielding aMUC1-PCVV (polymer-coated VV). PCVV infection was lower in cells expressing high amounts of MUC1, compared with uncoated VV; however, aMUC1-PCVV resulted in restoration of infection. These results demonstrated that binding of anti-VV neutralizing antibodies can be successfully and significantly decreased by both the targeted VV (aMUC1-PCVV) and the targeted VV’s unique chemical alteration. Additionally, both PCVV and aMUC1-PCVV, compared with bare VV, demonstrated enhanced evasion of the innate immune response.

Injectable polyvinyl alcohol microgels developed by combining microfluidics technology and a Michael type addition crosslinking reaction have achieved efficient loading of OAs and cancer virus therapy^138^. These pH-degradable microgels extend the survival period of OA in tumor tissues and increase the accumulation of tumor OA. The simultaneous delivery of JQ1 mediated by polyvinyl alcohol microgels has also been found to significantly inhibit PD-L1 expression, thereby overcoming immunosuppression during viral therapy.

Spain-based Sagetis Biotech is working to develop an innovative polymerization technology to create secure and efficient delivery methods—a key engineering barrier in gene therapy. VIROSHIELD™ coating technology alters the behavior of OVs and improves therapeutic efficacy through multiple means. The coated Ad protects against pre-existing neutralizing antibodies by masking its surface epitopes while retaining its elevated transfection capacity. In addition, lower activation of the adaptive immune response (3-fold decrease in neutralizing antibody production) and improved blood circulation time (3-fold increase) have been observed. The company has developed Senescence-associated gene 101 (SAG101), a polymer-coated Ad currently in preclinical studies, for the treatment of pancreatic ductal adenocarcinoma^139^.

### Application prospects

The use of OVs is limited by toxic adverse effects and neutralizing antibodies against OVs, as well as autoimmune clearance limits. A demonstrated strategy is to ensure that the drug is efficiently enriched at the lesion site, while decreasing the accumulation of the drug in normal tissues and organs. The rapid development of polymer nanocarriers has provided a viable approach to these problems. Currently, despite major advances in polymer nanocarriers, only several have been tested in preclinical animal *in vivo* models, and none have achieved clinical applications, primarily because of the lack of adequate means for in-depth assessment of the safety, biocompatibility, and degradability of novel polymer materials.

Although PEG has been widely used to improve the pharmacokinetics of protein drugs, increasing evidence indicates that prolonged use of PEG can cause the body to produce antibodies that considerably decrease treatment efficacy. ​For the polymer nanocarriers themselves, the complexity of structural design and the tediousness of material synthesis have also limited the industrial-scale production of these “smart” nanocarriers. Thus, researchers are motivated to continually develop and improve ideal biomedical polymer materials, and to explore carrier structure designs that are more amenable to clinical translation.

## Albumin

Albumin is the single-chain polypeptide with the highest content in the human body (normally accounting for more than 50% of the total plasma protein content), and it comprises 585 amino acid residues. Albumin is one of the smallest proteins present in the plasma, with a molecular weight of approximately 67 kDa^140^. Amounts of 13 to 14 g of albumin are produced in the liver and reach the bloodstream daily. Natural recycling mechanisms transport albumin from tissues back to the circulatory space *via* the lymphatic system^141^. Moreover, albumin is a generic macromolecular carrier that facilitates the systemic circulation of a variety of endogenous substances with limited solubility, such as fatty acids and bilirubin. Additionally, albumin can bind drugs and consequently affect their biodistribution, bioactivity, and metabolism. Examples of these drugs include taxanes, sulfonamides, penicillins, and benzodiazepines^142^. Because of the above functions, albumin has been widely demonstrated clinically as a safe biomaterial for the design of drug delivery systems.

### Advantages and disadvantages of albumin as a nanomaterial for drug delivery

Compared with other carriers, albumin as a carrier of antitumor drugs has the following advantages. 1) Excellent biocompatibility: Albumin is an endogenous substance in the human body that does not cause toxic reactions, trigger autoimmune reactions, or cause adverse reactions, such as denaturation and degradation^143^. 2) Unique structure and properties: Albumin’s properties endow it with stability within a certain temperature and pH range. Therefore, for most exogenous substances, albumin is an ideal carrier that can improve the stability of exogenous substances^144^. 3) Excellent drug loading performance: Albumins have a distinct spatial structure and can encapsulate drugs in the form of physical encapsulation or chemical bond coupling. Studies have indicated that albumin increases the solubility of hydrophobic drugs in the plasma and has excellent protective effects toward easily oxidized drugs^145,146^. Pharmacokinetic studies have shown that albumin avoids recognition and phagocytosis by the reticuloendothelial system, and also passively targets organs such as the liver, kidneys, and bone marrow. Furthermore, it is covalently bound to the surface of albumin. 4) Modification of various substances with targeted functions (for example, chemical modification of amino groups in active lysines on the surface of albumin)^147^. 5) Longer half-life *in vivo*: Because albumin has a negative charge in the blood, macrophages have difficulty in clearing albumin. Therefore, long-term circulation can be achieved^148^. Together, these advantages of albumin have laid a foundation for albumin to serve as a useful drug carrier.

However, although albumin nanomaterials have excellent biocompatibility, nontoxicity, and nonimmunogenicity, they still have shortcomings in drug delivery: 1) they have a rapid degradation rate *in vivo* and are easily removed from the blood circulation; 2) they may react with proteins in the blood, thus decreasing uptake of nanomaterials by tumor cells^149^; and 3) because of their inherent structural properties, they may be unstable in the body’s environment, which contains various enzymes and protein complexes^150^.

### Advances in albumin-encapsulated OVs

**Figure 7:**
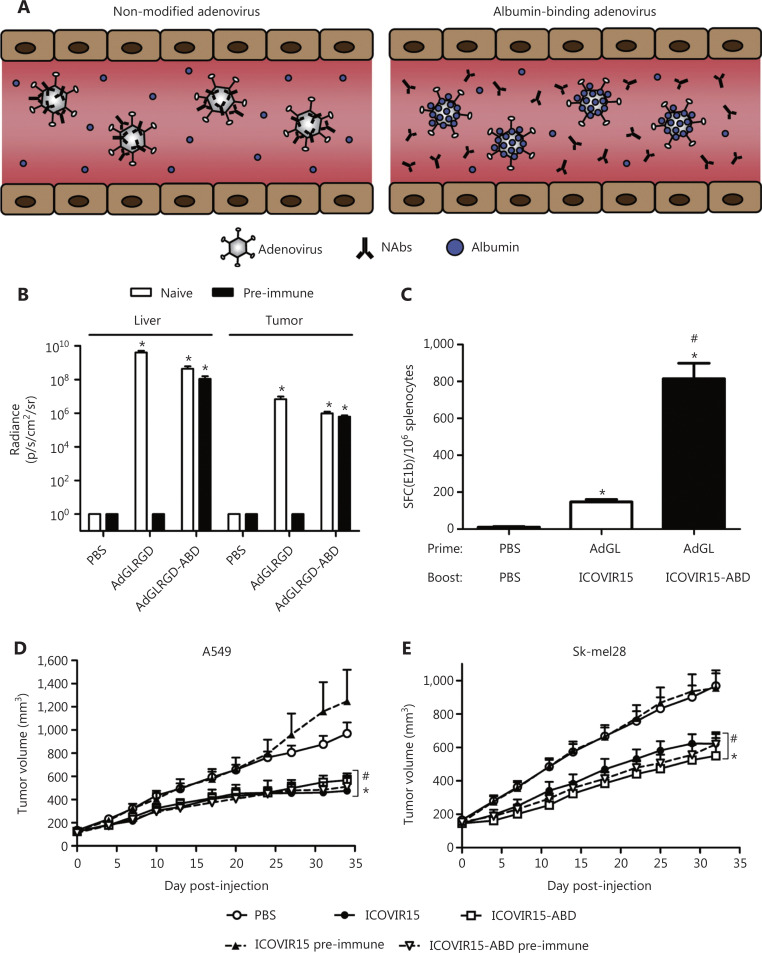
The albumin coating of the viral capsid effectively escapes pre-existing NABs. (A) Diagrammatic sketch. (B–E) ABD-modified adenoviruses evade NABs after systemic administration in pre-immune mice. Copyright © 2016 Elsevier B.V.

The binding of human and mouse albumin is mediated by insertion of an albumin-binding domain (ABD) into the hypervariable region 1 (HVR1) of the Ad capsid^151^ (**Figure 7**). After systemic viral treatment, albumin shields the capsid against NABs. The modified virus is totally neutralized in mice after Ad vaccination, and the ABD insertion safeguards the virus, thus enabling the same organ transduction and oncolysis as that observed in naive mice. Therefore, the ABD-mediated protection of viral capsids is an effective method to circumvent pre-existing NABs. This strategy has translational relevance in the use of adenoviruses for gene therapy, cancer virus therapy, and vaccination.

VCN-11, a novel Ad, has remarkable potential for eradicating of cancer cells. Ad VCN-11 was genetically modified to express hyaluronidase (PH20) and display ABD on the hexon. The virus can be coated with albumin, because of the presence of ABD, thus enabling avoidance of NABs as it enters the bloodstream. Using several tumor models, Mato-Berciano et al.^152^ have examined tumor targeting and anticancer activity in the presence of NABs. In tumor cells, compared with normal cells, VCN-11 showed a 450-fold greater cytotoxicity but less systemic toxicity. Thus, higher viral loads could be administered with a pre/post dosing technique, thereby improving tumor targeting. In addition, VCN-11 demonstrated efficient tumor targeting in the presence of high amounts of NABs *in vivo*, whereas control viruses without ABD were destroyed by NABs from various sources. Given these unique qualities, a proof-of-concept clinical trial is anticipated to analyze pre/post dosage fractionation techniques and repeat dosing, to determine the safety and tumor targeting abilities of VCN-11.

### Application prospects

In recent years, albumin has received substantial attention in nanodrug delivery systems, because of its excellent biocompatibility and low toxicity. The albumin nanodelivery system is characterized by extended circulation and tumor targeting, both of which significantly improve the therapeutic effects of the drug. In the future, an attractive research direction for albumin-based OV delivery systems is the design of environment-responsive albumin therapeutic systems. Tumor tissue has unique properties from those of normal tissue, such as an acidic environment, hypoxia, highly reactive oxygen species, and enzyme overexpression, all of which are fundamental for the study of responsive albumin nanotherapeutic systems. Albumin as a drug carrier has several drawbacks, such as limited sources of human serum albumin, a mild immune reaction to bovine serum albumin used in injections, and susceptibility to denaturation. Recombinant human serum albumin, a genetically engineered protein expressed by yeast cells, has been developed in recent years and can be used as an alternative to human serum albumin. The key to the clinical application of albumin nanofabrication is to identify stable raw materials and extremely reproducible preparation methods. An increasing number of albumin and OV-based drugs are expected to achieve clinical translation in the future.

## Other nanoparticles

In addition to the 3 types of nanoparticles described above, numerous biological techniques have been used for the systemic administration of OVs, including extracellular vesicles (EVs), encapsulation OVs, biomimetic mineralization of OVs, and hybrid carrier systems of OVs and organic framework materials. These biomaterials have excellent biocompatibility and *in vivo* targeted delivery performance, thereby broadening the range of applications for OV therapy for tumors.

### Advances in systemic delivery of extracellular vehicle-encapsulated OVs

EVs are membrane vesicles that are expelled by cells, and that transport proteins, carbohydrates, RNA, and DNA, thereby aiding in intercellular communication^153^. EVs have several benefits, including avoiding product identification and elimination in the body, crossing physiological obstacles, and having excellent biocompatibility and stability. The ability of these EV encapsulated medications to achieve tumor tropism is enabled by EVs’ susceptibility to artificial gene modification.

Using 2 separate biomimetic synthesis techniques, Lv et al.^154^ have created bioengineered cell membrane nanocapsules with tailored ligands to obtain significant antiviral immune shielding and targeting capabilities for oncolytic therapy. The second method is based on clustered regularly interspaced short palindromic repeats (CRISPR) engineering to express targeted ligands in red blood cell membranes *in vivo*. The first method uses *in vitro* genetic membrane engineering to embed targeted ligands into cell membranes. The findings have demonstrated that both bioengineering techniques preserve OA’s capabilities for infection and replication in the presence of NABs, in both *in vitro* and *in vivo* settings.

Ad5D24-CpG and paclitaxel (PTX) have been encapsulated in EVs by Garofalo et al.^155,156^, and found to significantly improve transduction efficacy *in vitro*, thus enhancing infection titer and cytotoxicity, and consequently anticancer effects in lung cancer models. Imaging technology has demonstrated that Ad encapsulated with EVs intravenously exhibits tumor tropism, whereas tumor selective delivery has not been observed through the intraperitoneal administration pathway.

To enhance the efficacy of cancer virotherapy by augmenting tumor cell autophagy, Ban et al.^157^ have used bacterial outer membrane vesicles (OMVs) containing Ads as microbial nanocomposites. These OMVs slow internal circulation clearance of OMV surface antigen and promote tumor formation by encasing itself in a biomineral shell. The catalytic activity of overexpressed pyranose oxidase (P2O) from microbial nanocomposites enhances oxidative stress levels and initiates tumor autophagy after tumor cell entry. The autophagy-induced autophagosomes further promote Ads replication in infected tumor cells, leading to Ads-overactivated autophagy. OMV, a potent immune stimulant, has been used in preclinical cancer models in female mice to alter the immunosuppressive tumor microenvironment and stimulate the anti-tumor immune response.

### Advances in biomineralized OV

OAs, because they preferentially reproduce in high titer tumor cells, provide an excellent option for clinical anticancer therapy. By encasing OAs in calcium carbonate and manganese carbonate (MnCaC) biomineral shells, Huang et al.^158^ have engineered an OA that prevents the immune system from clearing the virus, thereby prolonging its internal circulation. After assembling at the tumor site, MnCaCs dissolve and release Mn^2+^, thus resulting in conversion of endogenous H_2_O_2_ to oxygen (O_2_), increasing the ability of OA to replicate, and markedly increasing antitumor efficacy. A simultaneous rise in Mn^2+^ and O_2_ makes T1 mode magnetic resonance imaging and photoacoustic imaging feasible, and allows for real-time monitoring data in therapy.

## Conclusions and future perspectives

Intravenous delivery of OVs is a clinical issue that must urgently be addressed. Many preclinical studies have sought to determine the optimal delivery method, but no OV drug that can currently be administered intravenously to treat tumors. On the basis of these preclinical studies, we believe that liposomes, polymers, and albumins are the most promising nanomaterials to address these issues. Because many related drugs that have successfully entered the clinic have been packaged and delivered with liposomes, polymers, and albumins, their safety and practicality have survived clinical testing.

However, several critical issues in the delivery of OVs with these 3 nanocarriers must be addressed. The tumor microenvironment is extremely complex, and the targeting and therapeutic effects of nanomaterials are extremely limited. Because endogenous signals inside and outside tumor cells are difficult to control and vary among individuals, therapeutic effects in clinical practice are often not uniform. To address these problems, nanomedicine delivery systems with multiple targeting mechanisms can be designed. Unlike a single target, nanocarriers can carry 2 or more “guidance” signals simultaneously, thus improving the probability and accuracy of identifying patient tissues. In addition, bionic *in vitro* test platforms and preclinical models that can efficiently evaluate the performance of nanocarriers must be developed. Given the rapid advances in nanotechnology and the accumulation of relevant experience in recent years, we believe that these 3 newcomers will play an increasingly important role in the development of OVs. We also hope that continuous improvements will be made in personalized treatment approaches for OVs based on these 3 nanocarriers, to offer new hope in the treatment of malignant tumors.
